# Strain Modal Testing with Fiber Bragg Gratings for Automotive Applications †

**DOI:** 10.3390/s22030946

**Published:** 2022-01-26

**Authors:** Francesco Falcetelli, Alberto Martini, Raffaella Di Sante, Marco Troncossi

**Affiliations:** Department of Industrial Engineering-DIN, University of Bologna, Via Fontanelle 40, 47121 Forlì, Italy; francesco.falcetelli@unibo.it (F.F.); alberto.martini6@unibo.it (A.M.); marco.troncossi@unibo.it (M.T.)

**Keywords:** strain modal testing, optical fibers, fiber Bragg grating, strain frequency response function, carbon fiber reinforced polymers

## Abstract

Strain Modal Testing (SMT), based on strain sensors signal processing, is an unconventional approach to perform Experimental Modal Analysis which is typically based on data measured by accelerometers. SMT is still mainly restricted to academia and requires additional investigation for a successful transition towards industry. This paper critically reviews why the automotive sector can benefit from this relatively new approach for a variety of reasons. Moreover, a case study representative of the automotive field is analyzed and discussed. Specifically, an SMT methodology is applied to evaluate the modal properties of a reinforced composite roof belonging to a racing solar powered vehicle. In the experimental activity, signals from Fiber Bragg Grating (FBG) sensors, strain gauges, and accelerometers were simultaneously acquired and further processed. The advantages of using optical fibers were discussed, together with their weaknesses and ongoing challenges. The FBG results were compared with the conventional analysis performed with the accelerometers, emphasizing the main similarities and discrepancies.

## 1. Introduction

Nowadays, Experimental Modal Analysis (EMA) is still a fundamental tool in the design development [1]. It permits the assessment of the numerical model accuracy, the understanding of the vibration response level under operational conditions and the determination of certain material properties when the structure is subjected to dynamic loading [2]. In most cases, EMA relies on displacement, velocity, or acceleration measurements, and is referred to as Displacement Modal Testing (DMT). In DMT, modal parameters are estimated by means of the receptance matrix, namely the system Frequency Response Function (FRF) [2,3,4,5].

In the field of Structural Health Monitoring (SHM) [6], DMT can be used to identify changes to modal characteristics which can be linked to the presence of damage [7,8,9]. Despite DMT proving to be efficient as a global method, it is not the preferred solution for local damage detection [10]. In the last two decades, along with the advancements made in SHM, there has been an increasing interest in performing EMA, exploiting already available permanently installed strain sensors [11]. Indeed, strain-based measurements are more sensitive to local damage [10]. When strain rather than displacement data are collected, EMA takes the name of Strain Modal Testing (SMT) [12,13,14]. In SMT, traditional accelerometers are substituted with strain sensors, typically piezoelectric strain gauges (SGs) [15,16]. Accordingly, in the assessment of the modal parameters, the FRF is replaced by the Strain Frequency Response Function (SFRF).

There are several reasons that make SMT attractive compared to classic DMT. SMT allows the direct computation of the strain field distribution without applying any analytical or numerical procedure [16]. In specific locations, motion sensors are ineffective. For instance, in the vicinity of clamped or pinned boundary conditions, the only possibility is to acquire data using strain sensors [17]. The use of SGs is more appropriate when the sensor’s size and weight are of utmost importance [18]. Such cases are common for all those applications where the aerodynamic behavior must not be compromised by the sensor presence. In SMT, the sensor setup previously used during the production process development (usually SGs for remaining useful life estimations and fatigue testing) can also be exploited for the estimation of the structure modal parameters [19]. Zhou and Tao performed STM and exploited stress mode shapes to predict hotspots of multi-axial random fatigue [20]. In a recent article, Zhou et al. leveraged SMT for local structural dynamic modification [21,22] of thin plates with holes/notches [23].

Despite SMT proving to be a reliable solution for various applications, its implementation is still limited, especially in industry. Its widespread use is prevented by several aspects. For instance, its higher sensitivity to local damage makes SMT also more sensitive to structural local changes, making its use more challenging in the presence of complex geometries [14]. Different from FRFs, SFRFs show peaks which are inversely proportional to the square of the frequency [19], which implies that strain sensitivity is lower at the higher modes [14]. Other practical problems associated with SMT are the possibility of facing ground loops, more complex experimental setups and calibration procedures, and the possible bonding degradation which could induce amplitude loss or phase delay [15]. The comparison between DMT and SMT also brings to the fore the mass-normalization challenge. In SMT, both displacements and strain modal shapes are not mass normalized [12,13,17,24]. One solution is to perform mass normalization with the aid of an auxiliary motion sensor. However, in certain applications, this is not possible, resulting in unscaled modal shapes [17]. In Operational Modal Analysis (OMA), since the structure is excited by unknown ambient forces under operational conditions, analogous challenges are present [25,26]. Successful scaling has been achieved in OMA using mass-change strategies methodologies [27,28]. Kranjc et al., taking inspiration from the mass-change strategy already employed in OMA, developed a methodology to scale modal shapes in SMT. The authors demonstrated that with such methodology it was possible to match the results previously obtained using a Finite Element Method (FEM) [17]. Hence, SMT presents both advantages and disadvantages with respect to conventional DMT. 

In the last decade, the remarkable growth in several fields such as SHM, robotics and wearable sensors fostered the development of innovative flexible and stretchable force sensors for strain and pressure measurements (i.e., polymer/carbon nanotube-based sensors) [29]. These advancements may lead to innovative solutions in the field of SMT.

Similarly, the advancements in fiber optic technologies, and specifically the use of Fiber Bragg Grating (FBG) sensors, may tip the scales in favor of SMT. It is well-known that there are some benefits to using optical fiber in comparison with traditional electric strain sensors. Optical fibers are immune to electromagnetic fields, show high bandwidth, and have an excellent durability [30]. Moreover, they have been proven capable of measuring other measurands besides strain and temperature. A recent review article on optical fiber sensing for marine environment and marine SHM reported that optical fiber sensors can be employed to sense several chemical parameters, such as pH and heavy metals [31], as well as physical parameters such as salinity and pressure [31]. For example, extrinsic Fabry–Perot interferometric (EFPI) sensors have been successfully used for strain monitoring in high-temperature alloys and for pressure measurements [32]. Olyaee and Dehghani developed a two-dimensional photonic crystal sensor capable of performing highly accurate pressure measurements in the range from 0 to 2 GPa [33]. Lawson et al. used FBGs and developed an EFPI sensor to perform strain and pressure measurements during in-flight operational conditions [34].

Optical fibers are light weight, which makes their effect on the structure under test negligible. Moreover, their small size allows them to be embedded in composite materials, which are now becoming the new standard in many automotive applications [35,36]. In the manufacturing process of composite components, FBG can serve as a tool to monitor the curing process and the residual stain arising at the end of it [37]. During the testing phase, FBGs can be used to perform SMT, helping in the design development. Finally, once the manufacturing and testing phases are concluded, they provide a ready-to-use SHM system which can be used to make both diagnosis and prognosis of the structure.

Much research has been carried out to study the mechanical coupling, the fiber protection at the ingress-egress points, and the spectral response of embedded FBGs [30]. Recently, they have been successfully embedded in additive manufacturing specimens produced with fused deposition modeling technology [38,39]. In every embedding process, it is extremely important to consider the strain transfer phenomenon [40,41], which is responsible for the discrepancy between the measured strain at the fiber core and the real strain in the structure under test.

The aerospace industry can be considered the driving sector for the use of embedded FBGs in composite structures. They were employed for different purposes such as strain-based shape reconstruction, strain monitoring of wing structures, life-cycle monitoring of L-shaped components, debonding detection in lap joints and composite patches, damage detection in advanced grid structures, sensing elements in shape memory alloy-based control systems for smart composites, etc. [30]. Since the automotive industry is seeing a progressive transition toward composite structures, it is reasonable to expect an increasing use of FBGs also in this sector.

Distributed sensing technologies based on Rayleigh or Brillouin backscattering [42,43] are attractive since they offer a large number of sensing elements, but at the same time, their use in SMT is compromised by their limited sampling rate. On the other hand, FBGs can acquire signals at high frequencies (~1 MHz [30]), depending on the specific interrogation technique. Moreover, FBG sensors have a high multiplexing potential, which is fundamental for the monitoring of large structures. For example, FBG-based accelerometer sensors have been used to monitor the dynamic response of an offshore oil platform [44]. Hence, the combination of these characteristics made FBGs successful in a variety of engineering applications and also makes FBGs particularly suited for SMT.

Cusano et al. conducted a feasibility analysis to perform SMT by employing FBGs. They embedded the fibers in a composite wing of an aircraft model and derived the SFRF of system [45]. Peeters et al. applied SMT to the main rotor blade of a PZL W-3 helicopter. The authors bonded FBG sensors on the blade surface along with conventional electric SGs to compare the performance of the two systems [19]. Lamberti et al. analyzed the vibration properties of a carbon fiber reinforced control car arm using FBGs and validated the results taking as reference the measurements obtained with a laser Doppler vibrometer [46]. The same authors applied a similar methodology for the SMT of a glass fiber reinforced aeronautic hinge arm [47].

Despite the increasing interest in SMT using FBG sensors, the analysis of the literature suggests that further studies are required to understand the differences between DMT and SMT concerning accuracy and performance [16]. In this context, the aerospace field represents the driving force for the majority of these recent advancements, while only a few isolated studies come from the automotive sector [37,46,47].

This paper extends the work carried out in a previous research study [48] with novel experimental results, presenting in detail the concept of SMT and the FBG sensing principle. The ultimate aim of the study is to investigate how SMT compares to DMT and understand if FBG can be conveniently embedded in automotive applications, to monitor the modal parameters and their potential variations (simultaneously with the local strains), hence possibly enabling global vibration-based SHM for early fault detection.

The article is structured as follows. Section 2 reviews the theory behind STM and the sensing principle of FBG sensors. Section 3 presents the materials and methods applied in this study. Section 4 shows the results of the research, comparing the FRF and SFRF. In Section 5, the paper outcomes are interpreted and discussed. Finally, in Section 6, the main conclusions of the research are drawn, and future research needs are outlined.

## 2. Theoretical Background

### 2.1. Strain Modal Analysis

The governing equation for a system with n degrees of freedom is given by:

(1) $$\left[M\right]\left\{\ddot{x}\right\}+\left[C\right]\left\{\dot{x}\right\}+\left[K\right]\left\{x\right\}=\left\{f\right\}$$

where [M], [C] and [K] are the mass, damping and stiffness matrix, respectively. The vector of the applied forces is denoted as {f}, while {x} represents the system displacement vector. If proportional damping is assumed, the system response, {X(ω)} can be computed using Equation (2):

(2) {X(ω)}=[H]{F(ω)}

where [H] is the FRF (or receptance) matrix and can be expressed as follows:

(3) $$\left[H\right]=\overset{m}{\underset{r=1}{\sum }}\Lambda_{r}^{-1}\left\{\phi_{r}\right\}{\left\{\phi_{r}\right\}}^{T}=\left[\Phi \right]{\left[\Lambda \right]}^{-1}{\left[\Phi \right]}^{T}$$

Each mode shape $\left\{\phi_{r}\right\}$, participates in the overall response of the system [49]. In Equation (3), [Φ] represents the displacement modal matrix, the superscript T denotes the matrix transpose operator, and [Λ] is a diagonal matrix which can be written as:

(4) $$\left[\Lambda \right]=\mathrm{diag}\left(\omega_{r}-\omega^{2}+2{j\xi }_{r}{\omega \omega }_{r}\right)$$

where $\omega_{r}$ and $\xi_{r}$, are the rth modal frequency and the rth modal damping ratio, respectively. The letter $j=\sqrt{-1}$ represents the imaginary unit and ω is the excitation angular frequency. One strategy to derive the SFRF matrix is to introduce the S operator:

(5) $$S=\frac{1}{2}\left(\nabla +\nabla^{T}\right)$$

where ∇ denotes the linear differential operator. Applying the S operator to Equation (2) one obtains:

(6) $$\left\{X^{\epsilon }\left(\omega \right)\right\}=\left[H^{\epsilon }\right]\left\{F\left(\omega \right)\right\}$$

where $\left\{X^{\epsilon }\left(\omega \right)\right\}$ denotes the strain vector response of the system and $\left[H^{\epsilon }\right]$ is the SFRF matrix, which is computed using Equation (7):

(7) $$\left[H^{\epsilon }\right]=\overset{m}{\underset{r=1}{\sum }}\Lambda_{r}^{-1}\left\{\psi_{r}\right\}{\left\{\phi_{r}\right\}}^{T}=\left[\Psi \right]{\left[\Lambda \right]}^{-1}{\left[\Phi \right]}^{T}$$

where $\left\{\psi_{r}\right\}$ symbolizes the rth strain mode vector and [Ψ] is the corresponding strain modal matrix. Comparing Equation (3) with Equation (7), it is possible to see that, different from the FRF [H], the SFRF matrix is not symmetric, implying that the reciprocity principle does not hold in SMT.

### 2.2. Fiber Bragg Gratings

The history of Fiber Bragg Gratings (FBGs) dates to 1978, when Hill et al. observed index of refraction changes in germanium-doped silicate fibers [50]. In 1989, Meltz and coworkers made a breakthrough in the field, proposing a new methodology to generate FBGs using coherent UV radiation [51]. Since then, FBGs fabrication technology attracted the interest of many researchers [52], and there is a wealth of literature describing its evolution [53,54,55].

**Figure 1:**
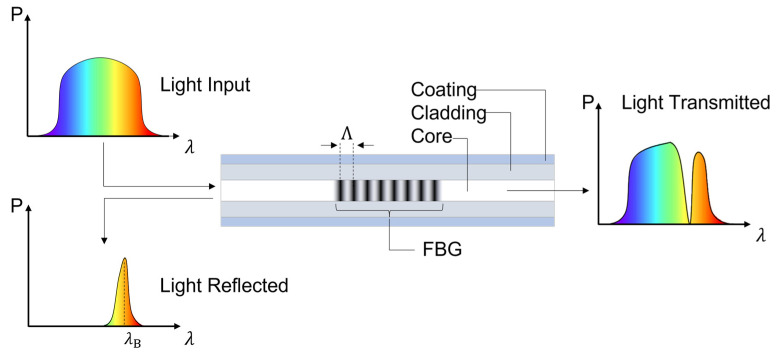
FBG working principle. In the transmitted spectrum, it is missing the power (P) associated with the Bragg’s wavelength, which can be measured in the reflected spectrum.

Figure 1 summarizes the FBG working principle. The grating visible at the center of the fiber can be regarded as a periodical refractive index modulation. Although the refractive index variation is oftentimes illustrated as a square waveform, its shape is better represented by a sinusoidal function [55]. Uniform FBGs (i.e., having constant index modulation and grating period) show undesired sidelobes in the reflected spectrum, which can be reduced through proper apodization profiles of the refractive index [56,57,58]. Monotonically varying the grating period will lead to chirped FBGs [59], whereas tilting the grating planes from their original orthogonal direction with respect to the fiber longitudinal axis will produce a tilted FBG [60].

This section provides a brief mathematical description for uniform FBG sensors. According to the Bragg’s law, when a broadband incident optical field illuminates an FBG, which can be regarded as the periodical effective refractive index ($n_{\mathrm{eff}}$) variation of period (Λ), only the Bragg’s wavelength ($\lambda_{B}$) is reflected, leading to the following well-known equation [61]:

(8) $$\lambda_{B}=2n_{\mathrm{eff}}\Lambda$$

When the FBG is subjected to longitudinal strain (ε), its period changes, which in turn produces a shift in the Bragg’s wavelength (${\Delta \lambda }_{B}$) [62]:

(9) $$\frac{{\Delta \lambda }_{B}}{\lambda_{B}}=\left(1-p_{e}\right)\varepsilon$$

where $p_{e}$ represents the effective photo-elastic constant and can be expressed as a function of the Poisson’s ratio (ν) and the Pockel’s coefficients $p_{11}$ and $p_{12}$:

(10) $$p_{e}=\frac{n_{\mathrm{eff}}^{2}}{2}\left[p_{12}-\nu \left(p_{11}+p_{12}\right)\right]$$

For FBGs written in standard optical fibers with a Bragg’s wavelength of 1500 nm, the expected sensitivity value is of 1.2 pm/με [63]. Another fundamental figure of merit is the detection limit, which can vary according to the interrogation technique. The interested reader can find typical detection limits values in the work of Campanella et al. [64].

The effect of temperature can be estimated by differentiating Equation (8) as follows:

(11) $${\Delta \lambda }_{B}=2\left(n_{\mathrm{eff}}\frac{\partial \Lambda }{\partial T}+\Lambda \frac{\partial n_{\mathrm{eff}}}{\partial T}\right)\Delta T$$

Equation (11) can be reshaped as [54,65]:

(12) $${\Delta \lambda }_{B}=\lambda_{B}\left(\alpha +\frac{1}{n_{\mathrm{eff}}}\frac{\partial n_{\mathrm{eff}}}{\partial T}\right)\Delta T=\lambda_{B}\xi \Delta T$$

where α is the thermal expansion coefficient of the optical fiber (e.g., silica), which summed to the thermally induced effective refractive index change, leads to the thermo-optic coefficient ξ. For a germanium-doped silica optical fiber, the effect of temperature on the wavelength shift is mainly dominated by the consequent change in the refractive index, which is in the range of $5-10\cdot 10^{-6}\;{}^{\circ}C^{-1}$, rather than the inherent thermal expansion of the optical fiber, since α is approximately $5.5\cdot 10^{-7}\;{}^{\circ}C^{-1}$ for silica. Applying these values to equation 12, with a Bragg’s wavelength of, it is possible to compute the sensor sensitivity with respect the temperature, which happens to be between $8-16\cdot 10^{-6}\mathrm{pm}/{}^{\circ}C$.

If both strain and temperature effects are present simultaneously and assuming independency between the strain and the thermal response, which holds for small perturbations [62], the change of the Bragg’s wavelength can be expressed using Equation (13) [63,66]:

(13) $${\Delta \lambda }_{B}=K_{\varepsilon }\Delta \varepsilon +K_{T}\Delta T$$

where $K_{\varepsilon }$ and $K_{T}$ are strain- and temperature-related constants whose values can be computed experimentally.

## 3. Materials and Methods

This section shows the experimental methodology employed in this study. First, the multi-sensors experimental setup is shown, explaining the reasons for the choices that were made. Second, the synchronization procedure required to perform SMT when using both optical and electrical based interrogators is showcased.

### 3.1. Experimental Setup

The structure under test was developed within the ONDA SOLARE^®^ project [67], coordinated by the University of Bologna (Italy). This project consisted in the design of a solar powered electric vehicle with the ultimate goal of competing in the American Solar Challenge competition in the Multi-Occupant Vehicle category [68,69]. The initial design was readapted to meet the World Solar Challenge regulations [70,71]. The solar vehicle has a length of 4610 mm, a width of 1775 mm and a height equal to 1230 mm [69]. The vehicle mass is 300 kg, thanks to the extensive use of Carbon Fiber-Reinforced Plastic Polymer (CFRP), and the payload, consisting of the four occupants, is estimated to be 320 kg. The vehicle is equipped with 64 kg lithium–ion batteries, charged by a 5 m^2^ monocrystalline silicon photovoltaic panel, which is placed on the roof [72].

In this study, the structure under test is the roof of this solar vehicle, whose topology optimization was accomplished using a multi-step and -objective design approach, which led to a CFRP quadridirectional grid structure with a thickness of 5.2 mm [73].

**Figure 2:**
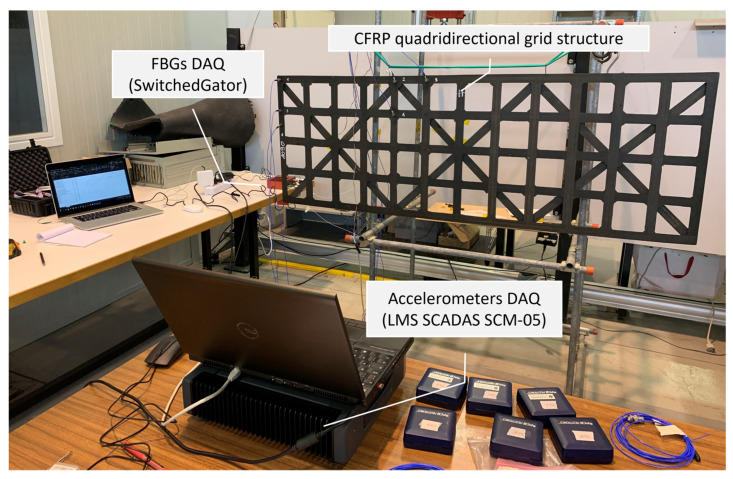
Experimental setup overview.

**Figure 3:**
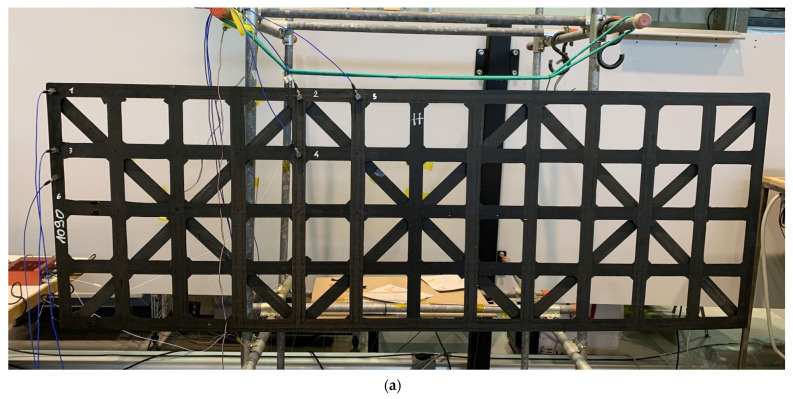

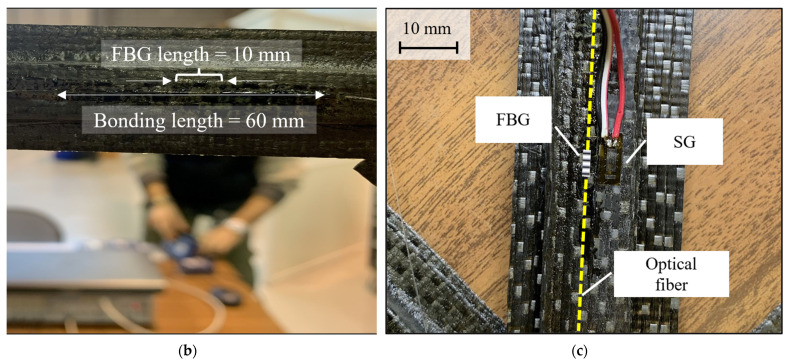
Experimental setup details: (**a**) Front view of the roof CFRP quadridirectional grid structure; (**b**) Example of FBG bonding; (**c**) Electrical SG bonding in proximity to FBG 8.

Figure 2 and Figure 3 show the experimental setup used in this study. The Carbon Fiber-Reinforced Polymer (CFRP) roof was suspended in a free-free configuration, and tested taking as reference the ISO 7626-5:2019 [74].

An impact hammer (model: 086C04) and six Integrated Circuit-Piezoelectric (ICP^®^) accelerometers (models: 333B32, 333B35, 352C03), which are a specific type of Integrated Electronic Piezoelectric (IEPE) accelerometers, from PCB Piezotronics (Depew, NY, USA) were employed to perform DMT. Data were acquired with an LMS SCADAS SCM-05 Data Acquisition System (DAQ), from Siemens AG (Munich, Germany), using LMS Test.Lab as software. The DAQ was set to acquire data with a sampling rate of 1024 Hz with a signal time duration of 4 s. This led to a frequency bandwidth of 512 Hz and a frequency resolution of 0.25 Hz. Considering the strain propagation time from the material to the accelerometers to be negligible, the corresponding response time is of 0.98 ms.

The structure was equipped with 8 uniform FBGs (see grating dimensions and bonding details in Figure 3b) divided into two different arrays (each one consisting of 4 FBGs) in order to perform SMT. The FBG spectrum was analyzed using the SwitchedGator [75] interrogation system from Technobis, now PhotonFirst (Alkmaar, The Netherlands). This interrogation unit is capable of handling 64 FBGs, multiplexed into 8 different channels and has a sampling speed of 19.23 kHz. The SwitchedGator has a minimum amount of residence time per channel of 2 ms and takes 1 ms for the switch operation. In this experimental activity, since two channels were employed, the time interval between two subsequent measurements was 3 ms. The time taken from a strain waveform to propagate from the material toward the gratings in the fiber core is expected to be some orders of magnitude lower than 3 ms. Therefore, the response time can be fairly approximated to 3 ms, leading to an effective sampling frequency of 333 Hz. The interrogator can acquire wavelengths in the interval 1515–1585 nm and requires a minimum spacing of 9 nm between two adjacent wavelengths (i.e., two adjacent FBGs). In this specific case, the FBGs wavelengths ranged from 1531 nm to 1560 nm; accordingly, the strain sensitivity was in the interval 1.18−1.24 pm/με. The structure was also equipped with an electrical SG in order to synchronize the optical fiber system with the accelerometer DAQ (details in Section 3.2).

**Figure 4:**
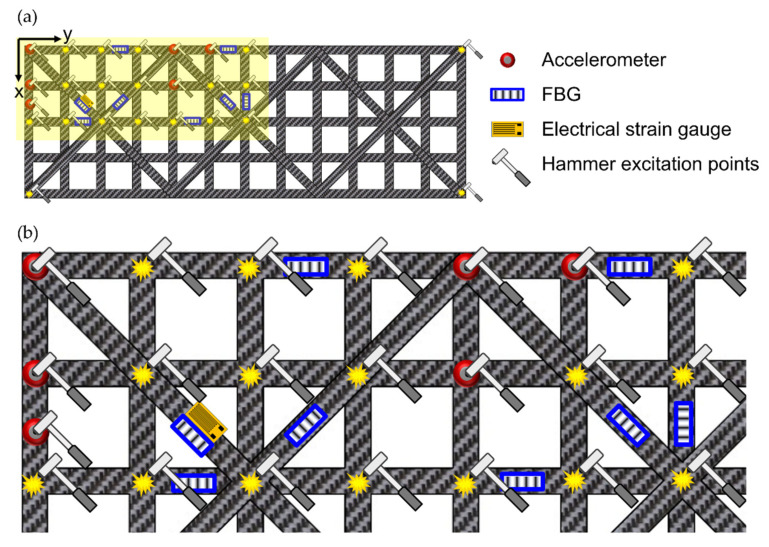
Sensor and hammer excitations: global view (**a**) and zoom view of the north-west corner (**b**).

Sensor placement was optimized by visually inspecting the mode shapes of interest by means of a previously developed FEM model of the CFRP roof [73,76]. The analysis considered the first six modes, whose natural frequencies were below 150 Hz. The accelerometers and the FBGs were placed in the locations with the highest values of displacement and strain, respectively. At the same time, particular attention was posed to avoiding the displacements nodes (accelerometers) and the strain nodes (FBGs) present in the first seven modal shapes. In lightweight structures, such as the one analyzed in this study, the additional mass caused by sensors installation should be considered. Despite the FBG’s weight being negligible, the accelerometers may have an impact on the modal response of the structure. These considerations bring to the fore the intrinsic compromise of keeping the number of sensors as low as possible without losing the spatial resolution required to avoid the space aliasing of the modal shapes. Therefore, sensors and hammer impact locations were selected leveraging the horizontal and vertical symmetries of the structure, which led to the configuration illustrated in Figure 4a. Except for the hammer impacts performed in the extreme north-east, south-east and south-west corners, all the sensors and hammer excitations were concentrated in the north-west quarter of the structure, as shown in Figure 4b.

At every excitation point, hammer impacts were repeated five times and subsequently post-processed to reduce noise and improve the signal to noise ratio.

Table 1 contains all the coordinates of the sensor layout used in this study, using the coordinate system shown in Figure 4a. The abbreviations “Acc”, “FBG”, “SG” and “Ham” were employed to indicate accelerometers, FBGs, electrical SG and hammer impacts, respectively.

The experimental activity was carried out in the laboratory at constant room temperature to make thermal effects negligible. Nevertheless, in the potential scenario of real time monitoring during operational conditions, additional considerations are required. The CFRP roof of a solar racing vehicle is expected to experience severe temperature gradient and fluctuations, especially in the skin of the structure, where the FBGs are installed. This would require a temperature compensation strategy. Temperature effects can be discriminated from pure mechanical strain using a multitude of different approaches [77,78,79,80,81,82,83,84,85,86,87]. Therefore, this aspect does not imply any loss of generality of the methodology presented in this study.

Finally, in real-time monitoring applications, the integration of the FBGs readout circuitry should be carefully addressed, respecting all the design constraints. However, these considerations are outside the scope of this study and will not be discussed in this article.

### 3.2. Synchronization Procedure

In DMT, the output of the accelerometers is synchronized with the input signal generated by the impact hammer. This synchronization is crucial for the derivation of a correct FRF. Most of the time, this requirement in DMT is assumed to be satisfied a priori since both the accelerometers and the impact hammer are connected to the same DAQ.

On the other hand, FBGs have their own DAQ. Trigger options are often only available for specific interrogators, making it difficult to ensure synchronization directly at the hardware level. The SwitchedGator, which was the DAQ used in this study to acquire FBGs signals, cannot be triggered by an external device and would require some custom modifications for this purpose. Therefore, the authors decided to follow an alternative synchronization methodology, already introduced by Peeters et al. in 2014 [19], which does not require a trigger in the optical DAQ. Specifically, an electrical SG was bonded in proximity to FBG 8 (see Table 1 for coordinates), as shown in Figure 4b, and acquired by the DAQ used for the DMT. This post-synchronization assumes that the electrical SG and the FBG sensor would have the same exact response. Since they cannot be installed one above the other, this hypothesis can be true only theoretically. However, if the sensors are sufficiently close to each other, their signals would be similar enough to be properly synchronized.

During post processing operations, the FBG 8 and electrical SG signals were resampled such that they could share the same sampling frequency. Then, signal synchronization can be achieved by performing the cross-correlation function of the two signals and determining the location of its peak. Alternatively, it is possible to synchronize the received signals by determining the distance between specific points of interest, such as maxima or minima. In this specific case, the latter approach was used since it proved to be more robust against noise than the former.

## 4. Results

In this section, the main results of this research are presented. First, in Section 4.1 the synchronization plots are shown. Then, in Section 4.2 the FRF and SFRF are compared. The total amount of recorded and post-processed signals in this study is summarized in Table 2.

### 4.1. FBG vs. SG

**Figure 5:**
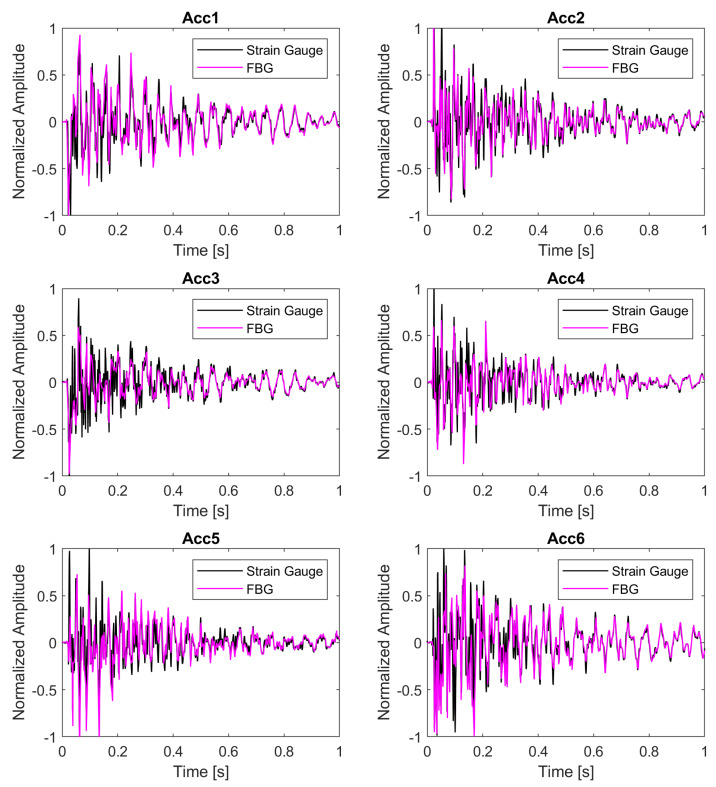
Examples of synchronization plots between FBG 8 and the SG.

The synchronization between the electrical and optical systems was carried out aligning the SG and the FBG 8 signals for every excitation performed with the roving hammer. As an example, Figure 5 reports the synchronization plots of the sensor responses corresponding to one (out of five) hammer impacts exciting the structure at the six accelerometer locations. As expected, the SG and FBG 8 signals are not identical. Nevertheless, they are in good agreement, allowing the alignment of the FBG signals, acquired using the SwitchedGator, with the hammer impact signals, which were recorded with the LMS SCADAS SCM-05 DAQ.

Further post-processing was performed by utilizing the MATLAB software (MathWorks, Natick, MA, USA). The first step consisted of computing the auto-power and cross-power spectral density of the input and output signals using the Welch’s method. Successively, the *H1*, *H2* and *Hv* estimators were computed to determine the SFRFs. Moreover, the coherence functions were derived in order to assess the SFRFs quality. Preliminary results showed that the *H1* estimator was the most appropriate for the analysis. This is in accordance with the fact that *H1* is an unbiased estimator in the presence of noise in the output signal [88], which in this case is expected to be higher than the noise in the input signal. Thus, the *H1* estimator was utilized to compute and plot the SFRFs shown in this study.

**Figure 6:**
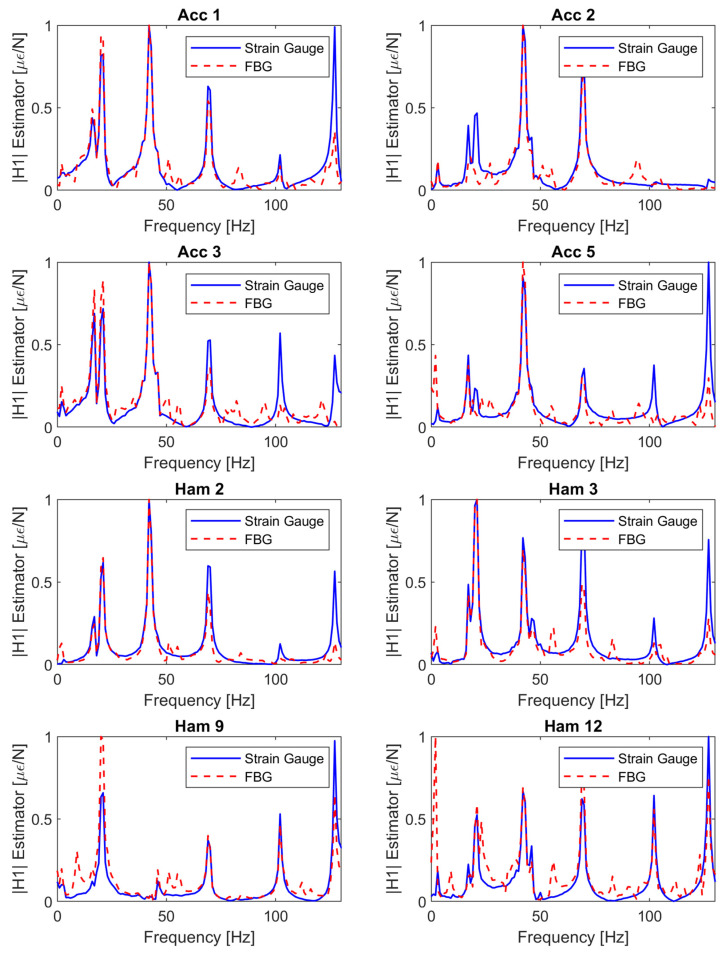
Electrical SG and FBG SFRF comparison at several hammer excitation locations.

Figure 6 shows the comparison of FBG 8 and the electrical SGs in terms of SFRF at several excitation locations (see Table 1 for coordinates). All the plots suggest that there is a substantial agreement between the FBG and SG SFRFs computed with the *H1* estimator.

### 4.2. FRF vs. SFRF

This section reviews the results concerning the comparison between the SFRFs obtained with the FBGs and the FRFs derived using the traditional accelerometers. The FRFs were initially computed directly inside the LMS Test.Lab software environment, exploiting its internal algorithm for the *H1* estimator. Nevertheless, it was decided to perform a preliminary analysis by computing the same FRFs with the same MATLAB algorithm employed to derive the SFRFs. The two approaches returned equivalent results, proving the exactness of the MATLAB script developed by the authors. This intermediary step was important to discern the algorithm-induced differences from the real dissimilarities of FRFs and SFRFs.

**Figure 7:**
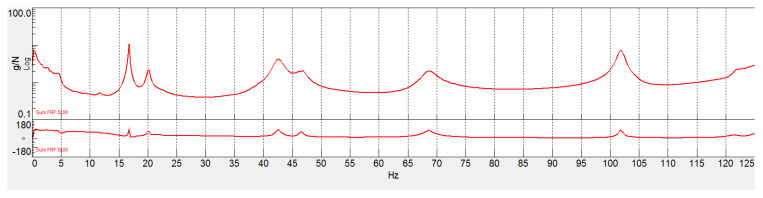
FRF sum resulting from accelerometer data.

Figure 7 shows the sum of all the FRFs computed with the accelerometers performed in LMS Test.Lab. For the real part, the sum is computed considering the absolute value of the real part of every single FRF. The same procedure is applied to the imaginary parts. This method to compute the average FRF is useful because it avoids the possibility that the contribution of positive and negative peaks is lost during the summation process.

**Figure 8:**
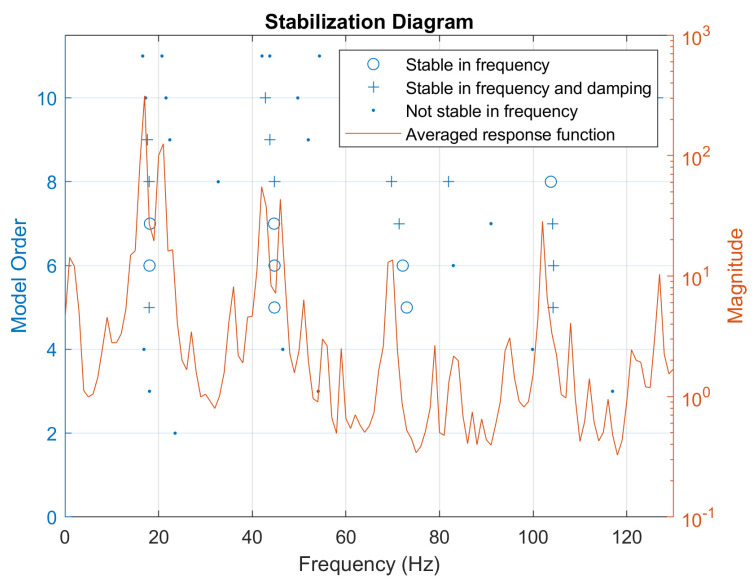
SFRF stabilization diagram of FBG data.

On the other hand, Figure 8 shows the stabilization diagram computed in MATLAB making use of the FBG data. The plot is produced using the *modalsd* function which exploits the Least-Squares Complex Exponential (LSCE) algorithm.

Both graphs provide similar information, even if extra noise is present in the optical fiber plot. Moreover, the results were compared with preliminary available FEM results [73], showing a satisfactory agreement between experimental data and numerical results (obtained from the first FEM model, non-validated), with the only exception being the sixth mode, as reported in Table 3 where percentile variations are computed with respect to the DMT results, taken as reference.

## 5. Discussion

The results presented in Figure 6 showed that FBG 8 and the electrical SG produced similar SFRFs at several excitation points. The similarity in the results suggests the consistency of the FBG coupling with the structure, which can be undermined by a not adequate strain transfer. Discrepancies consist mainly in extra peaks present in the FBG SFRFs. However, the comparison with the stabilization diagram of Figure 8 suggests that these differences have a numerical nature and do not imply extra physical modes detection from the FBGs, confirming the consistency of the results.

Figure 7 and Figure 8 highlighted that SMT conducted with FBGs produces analogous results with respect to DMT performed with traditional accelerometers. Differences in the peaks’ amplitude and position can be associated with the different location of FBGs and accelerometers. This configuration depends on the case study, but it is also likely to be true for other applications. Indeed, most of the time, displacements and strain nodes or maxima do not coincide. For example, in a cantilever beam, the displacement is expected to be maximum at the opposite end of the fixed joint, right in the place where the strain is expected to be null. Therefore, since strain and displacement sensors cannot be exactly in the same locations, it is logical to assume that the former set of sensors may be less sensitive to certain modes with respect to the latter and vice versa.

Moreover, from the analysis of the stabilization diagram in Figure 8, it is possible to infer that the extra peaks present in the SFRF function do not correspond to real modes since they are not deemed stable from the LSCE algorithm. Hence, the stable peaks of the stabilization diagram in Figure 8 coincide with the modes identified in Figure 7.

In both Figure 6 and Figure 8, the extra amount of noise present in the FBGs response can be reasonably attributed to the synchronization procedure which, despite being successful, introduced noise into the system, leading to a lower signal to noise ratio.

The impossibility to install the FBG and the SG exactly in the same location (see Figure 3c), is a limiting factor to achieve a perfect synchronization. Another reason is associated with the sampling frequencies of the two DAQs. Figure 5 reports the acquired raw signals after the synchronization procedure. Discrepancies between the SG and the FBG are larger near the initial measurements. The SwitchedGator acquires data at a lower frequency (333 Hz) with respect to the LMS SCADAS SCM-05 DAQ (1024 Hz). Hence, in the initial portion of the signal, the spectrum is dominated by high frequencies, which are captured only by the SG. On the other hand, in the second part of the signal, the high frequencies are damped and the agreement between the FBG and the SG increases.

Whether this uncertainty is acceptable or not depends on the specific application.

This study proves that if synchronization at hardware level is not an option, the results are still consistent even though there is a slight degradation in the signal quality of the optical system. In future studies, this aspect might be further investigated comparing the results of data synchronized with a trigger in the interrogator with other results synchronized during the post-processing phase following the procedure adopted by Peeters et al. [19], and in the present study.

## 6. Conclusions

In this study, SMT using FBGs was compared with DMT employing conventional accelerometers in a reinforced CFRP roof of a solar powered vehicle. Sensor placement was carried out by exploiting the symmetries of the structure and using preliminary knowledge from a numerical model.

The accelerometers were acquired with a sampling frequency of 1024 Hz, whereas FBGs having a strain sensitivity higher than 1.18 pm/με were acquired at 333 Hz. The FBGs’ sensitivity to temperature was neglected because experiments were conducted at constant ambient temperature. In real operational conditions, temperature variations are expected to modify the FBGs output which can be compensated using adequate compensation schemes. However, the temperature fluctuations are expected to occur at frequencies lower than the ones associated with the dynamic response of the structure, implying that their effect should be negligible in any case.

SMT and DMT provided comparable results, which is confirmed by the percentage differences in the estimated modes frequency always below 5% (Table 3). Using both methodologies, it was possible to identify the expected modes in the frequency bandwidth of interest predicted by preliminary FEM results.

Although SMT requires a synchronization procedure between the FBGs and impact hammer DAQs, which increases the complexity of the overall methodology, there are clear benefits which make SMT preferable to DMT.

The key advantage of employing FBGs for modal analysis, rather than accelerometers, lies in their multi-purpose capabilities. Specifically, they allow use of both local and global damage detection schemes at the same time. On the one hand, the use of permanently installed FBG sensors has already proven to be useful for the stress/strain monitoring of relevant structural locations (hot-spot monitoring). This local damage detection functionality can be used in the manufacturing process (for example, to monitor the curing process), in the design phase (testing of mechanical properties), and in operational conditions (real-time damage detection). On the other hand, since the presence of damage is expected to alter the modal properties of the system (hence allowing early failure detection), the FBGs network can also be employed to perform vibration-based global damage detection through SMT.

In conclusion, FBGs proved to be adequate to perform SMT, which can be potentially adopted across different structural health monitoring fields (automotive, aerospace, marine, etc.). In the current state of the art, SMT through FBGs for automotive applications has been reported only in a few case studies and is at its infancy. FBGs’ benefits are clear, and their use in the automotive industry is expected to increase in the coming years. The authors hope that the present study may promote further research investigations in this field, which is essential for a complete transfer of this technology from academia towards the automotive industry.

## Figures and Tables

**Table 1:** Coordinates of sensor locations and hammer impact nodes.

Sensor	X (mm)	Y (mm)
Acc 1 ^1^	15	15
Acc 2 ^1^	15	530
Acc 3 ^1^	145	15
Acc 4 ^1^	145	530
Acc 5 ^1^	15	655
Acc 6 ^1^	210	15
FBG 1	200	785
FBG 2	270	600
FBG 3	200	720
FBG 4	15	720
FBG 5	270	200
FBG 6	200	340
FBG 7	15	340
FBG 8	200	200
SG	200	200
Ham 1	530	15
Ham 2	530	1550
Ham 3	15	1550
Ham 4	145	270
Ham 5	270	15
Ham 6	15	140
Ham 7	145	140
Ham 8	270	140
Ham 9	15	270
Ham 10	270	270
Ham 11	15	400
Ham 12	145	400
Ham 13	270	400
Ham 14	270	530
Ham 15	145	655
Ham 16	270	655
Ham 17	15	785
Ham 18	145	785
Ham 19	270	785

^1^ The hammer impacts performed in correspondence of the accelerometer locations are not reported in the table to avoid the repetition of the same set of coordinates.

**Table 2:** Summary of post-processed signals.

Excitation Points	Repetitions	Hammer Impacts
25	5	25×5=125
**Sensor Type**	**Number**	**Sensor Signals**
Accelerometers	6	125×6=750
Fiber Bragg Gratings	8	125×8=1000
Strain gauge	1	125×1=125
		**Tot. Post-processed signals** 125+750+1000+125=2000

**Table 3:** Natural frequencies comparison.

Mode	Accelerometers [Hz]	FBGs (Hz (%))	FEM (Hz (%)
I	16.7	17.0 (+1.8%)	15.3 (−8.4%)
II	20.1	21.0 (+4.5%)	18.7 (−7.0%)
III	42.6	42.0 (−1.4%)	40.1 (−5.9%)
IV	46.5	46.0 (−1.1%)	41.7 (−10.3%)
V	68.6	69.5 (+1.3%)	65.4 (−4.7%)
VI	101.8	102.0 (+0.2%)	81.9 (–19.5%)

## Data Availability

The data presented in this study are available on request from the corresponding author.

## Abbreviations

The following abbreviations are used in this manuscript.

**Abbreviation**	**Meaning**
Acc	Accelerometer
CFRP	Carbon Fiber Reinforced Polymer
DAQ	Data Acquisition System
DMT	Displacement Modal Testing
EFPI	Extrinsic Fabry-Perot interferometric
EMA	Experimental Modal Analysis
FBG	Fiber Bragg Grating
FEM	Finite Element Method
FRF	Frequency Response Function
Ham	Hammer
ICP	Integrated Circuit-Piezoelectric
IEPE	Integrated Electronic Piezoelectric
LSCE	Least-Squares Complex Exponential
OMA	Operational Modal Analysis
SFRF	Strain Frequency Response Function
SG	Strain Gauge
SHM	Structural Health Monitoring
SMT	Strain Modal Testing
